# Opinion Leadership among Nurses in Public Debate: Moral Conviction, Credibility, and Collective Support

**DOI:** 10.1177/15271544261444730

**Published:** 2026-05-20

**Authors:** Daphne A. E. Spoolder, Jessica van Wijk, Dorine van Staalduinen, Pieterbas C.B. Lalleman

**Affiliations:** 1 Department for Research and Innovation, 6028 St. Antonius Hospital, Nieuwegein, Netherlands; 2 Erasmus University Rotterdam Erasmus School of Health Policy and Management, Research Group for Person-Centeredness in an Ageing Society, 113896 Fontys University of Applied Sciences, Rotterdam, Netherlands; 3 61363 Martini Academy, Martini Hospital, Groningen, Netherlands; 4 Research agenda, Santeon, Utrecht, Netherlands; 5 Department of Health Studies, The Research Group for Person-Centeredness in an Ageing Society, 3170 Fontys University of Applied Sciences, Eindhoven, Netherlands

**Keywords:** nurses, leadership, health care reform, public opinion, policy, workforce

## Abstract

Nurses’ involvement in public and policy debates is increasingly recognised as essential for shaping health care systems, yet their voices remain largely invisible. When nurses do engage publicly, they may act as public opinion leaders, using their professional expertise and credibility to influence policy discussions. Little is known, however, about how public opinion leadership (POL) manifests in practice and what motivates nurses to take on such a role. A proposed health care reform in the Netherlands, known as the BIG2 proposal, triggered an unexpected wave of nurses speaking up in national media and on social platforms. This study explores how POL manifested during this reform and what motivated nurses to raise their voices. A qualitative design was used, combining a thematic and abductive approach. Twelve semi-structured interviews were conducted with Dutch nurses who publicly responded to the reform through newspapers or social media. The findings show that nurses who engaged in POL acted from moral conviction and professional credibility, rather than formal authority. Their motivation stemmed from feeling misunderstood or undervalued, which evoked emotions such as frustration and anger. Responsibility, encouragement, and solidarity sustained their engagement. Nurses used social and traditional media to connect with peers, journalists, and politicians, making their leadership visible and collective. POL appeared as a collective process grounded in moral conviction, professional expertise, and mutual support. These findings highlight how nurses can extend their influence beyond clinical settings and contribute to shaping health policy debates.

## Introduction

The nursing workforce has been under pressure worldwide. The World Health Organisation estimates a shortage of 4.5 million nurses by the year 2030 (Boniol et al., 2022). The situation is similar in the Netherlands, where the government predicts a nationwide shortfall of 14,300 nurses in 2032 (Helder, 2023). To retain the existing and future nursing workforce, and to strengthen nurse well-being, it is essential to improve the quality of the nursing work environment. Nursing governance (Spoolder et al., 2024) and leadership (Silva et al., 2022) are shown to positively influence these conditions.

An important aspect of a healthy and sustainable work environment is the extent to which nurses are involved in decisions that shape their professional roles and the organisation of care. Actively engaging nurses in policy and decision-making processes that affect their practice has been recognized as a key component of professional empowerment and quality improvement (Buurman, 2020; Cunha et al., 2019; Verhoeven et al., 2024). This involvement can take place within health care organisations, but also at a broader societal or political level, where nurses may engage in discussions on policy and public opinion.

This broader engagement can be understood as aligning with the concept of public opinion leadership (POL). Van Wijk et al. (2022, pp. 75–76) define this concept as “the action of influencing public debate regarding policymaking processes by maintaining dynamic (social) networks, having a high sense of systemness, and being (clinically) credible, altruistic and accessible to peers and a wide variety of stakeholders.” In essence, POL refers to nurses using their professional expertise and credibility to influence how nursing issues are discussed in the public domain. Through such engagement, nurses can help shape health policy and contribute to collective professional visibility.

However, despite their expertise in health care, nurses remain largely invisible in public and policy debates (Mason & Ricciardi, 2025; Van Wijk et al., 2025a). Downs and Fiore-Lopez (2022) suggest that nurses may hesitate to engage in public or political advocacy, as such actions can feel unfamiliar or outside their traditional professional role. This hesitation, together with a lack of visible role models or organisational encouragement, may explain why nurses rarely assume POL roles. Little is known, however, about what motivates nurses to speak up when they do engage publicly.

In the Netherlands, the educational entry levels for nursing are Bachelor education of Nursing (BN) (Bouwes et al., 2023), Vocational education of Nursing (VN) (V&VN, 2016) and Diploma Nursing (DN), (Van der Kemp & De Kleijn, 2018). Theories about influencing the strategic direction of the organisation are included only in Bachelor education of Nursing (BN) (Schalkwijk et al., 2024; Van Kraaij et al., 2023). This contributes to differences in how nurses are prepared for policy involvement.

To address these differences, and in order to improve the situation in the Netherlands, the Dutch government proposed a legislative reform in 2017 concerning the Professions in Individual Health Care legislation (in Dutch: Wet op de Beroepen in de Individuele Gezondheidszorg, or BIG). The proposed law, known as BIG2, aimed to introduce a formal distinction between BN and VN nurses, while aligning DN with VN (Ministerie van Volksgezondheid, 2017). This would have formalised two groups: those with a bachelor education and those without. Although intended to clarify professional roles and enhance career development, the proposal evoked a wide range of responses and resulted in a nationwide uprising among nurses (Bruins, 2019; Felder et al., 2022).

The Netherlands thus witnessed unprecedented resistance (Bruins, 2019), with nurses emerging as driving forces in the public debate: appearing on national broadcast TV and radio programmes, and publishing opinion pieces in nursing journals and newspapers. By using traditional and social media as powerful tools for nurses to engage in health policy and politics (Gardner et al., 2020), their collective media engagement directly contributed to the government's withdrawal of the proposed law (FNV, 2019).

What made this situation unique was that nurses themselves became the leading voices in the debate, rather than being represented by managers or professional organisations. They organised online campaigns, appeared in national media, and directly addressed policymakers. Their actions reflected several of the characteristics of POL as described by Van Wijk et al. (2022). However, this phenomenon has received little empirical attention in the Dutch context. This limited empirical attention becomes especially relevant when considering how existing literature conceptualises public opinion leadership (POL).

Existing work on POL in nursing has primarily focused on conceptual and theoretical development, including integrative reviews and concept analyses (Van Wijk et al., 2022), with limited empirical examination of how POL manifests in practice. While related studies have drawn on qualitative insights into nursing leadership, advocacy, and media engagement, systematic empirical exploration of POL as enacted by nurses in concrete policy debates remains scarce. This study addresses this gap by examining how POL was expressed and experienced by nurses during a national health policy controversy.Against this theoretical background, the BIG2 case offers a unique opportunity to observe how POL unfolded in practice. Understanding why these nurses decided to act, and how they used public and political channels to make their voices heard, can deepen insight into how nurses express leadership beyond the clinical setting. This study therefore aims to explore how POL manifested among Dutch nurses during the BIG2 debate and what motivated them to engage in this role. By examining this case, the study contributes to a broader understanding of how nurses express leadership in the public arena and how such engagement can help strengthen the professional voice of nursing.

## Objective

The aim of this study was to gain insight into the types of behaviours nurses engage in when publicly active, to explore motivations for such behaviour, and identify nurse actions to broaden their impact as publicly active leaders.

## Methods

The study deployed a qualitative design with thematic analysis and abductive approach, with semi-structured interviews.

### Participants and Setting

Purposive sampling was used to recruit Dutch nurses, who raised their voice in public media regarding differentiated nursing practice between June and October 2019. While being pro or con differentiated nursing practices appeared to be on a continuum, participants were selected based on how they expressed themselves in public media rather than on formal organizational roles or hierarchical positions. We aimed to recruit a diverse group of nurses with diverse opinions and educational backgrounds. Participants were selected by searching with search terms ‘function differentiaton’ OR ‘BIG2’ in Dutch newspapers, using Nexis Uni (LexisNexis, n.d.), as well as on social media, such as Twitter (now X), Facebook and LinkedIn. Participants were suitable if they were nurses and spoke up in media regarding BIG2. Seventeen nurses were approached through their social media accounts. A brief description of the study was sent and the subject information sheet (SIS), complemented by a written consent form, was then sent by e-mail. One individual rejected participation due to time constraints. Interviews with four individuals were not pursued after thematic saturation was reached, with no new information emerging (Smith & Noble, 2014)**,** after twelve interviews.

### Data Collection

Data collection took place via semi-structured interviews in February and March 2020. Nine interviews were held face-to-face, and due to the COVID-19 pandemic, three interviews were held over the telephone. The interviews took approximately 45 min. Demographic data were collected verbally just before the interview. After participants signed the informed consent form or gave vocal permission over the phone, the semi-structured interviews were conducted. The interview guide was developed by the main researcher and based on the definition of POL, according to Van Wijk et al. (2022). The main themes of the interview guide included personal traits, actions in media, and perceived effects of speaking out, such as ‘What made you share your opinion in public media?’ (Table 1).

**Table 1.** Interview guide.

**Main Question** What were your reasons and motives for sharing your opinion in public media, with regards to the health care reform?
**Opening** I would like to talk about your media appearance* What was past summer like for you? What were your first thoughts about the law proposal?
**Personal traits of the opinion leader** What did you need to voice your opinion? Did you require certain skills to do this? Were you always like this? What changed?
**Action** What action did you take after hearing about the law proposal? What made you share your opinion in public media? What goal did you have? What part does media play in showing public opinion leadership? (television / radio / social media / newspaper, etcetera)
**Result** Did you receive many reactions? What were those reactions like? Did you receive any negative reactions? Did these reactions affect you? How are you now? Would you do share your opinion again? Do you think you have influenced politics or legislation? Was it all worth it? Did you reach your goal?
**Final question** You did (not) reach your goal, but you did voice your opinion. I would like to thank you for that. Did we forget to ask anything important?

*Sentence added after three interviews.

### Data Analysis

All interviews were transcribed and imported into QSR International's NVivo 12 qualitative data analysis software (QSR International Pty Ltd, 2018). The transcripts were recursively coded and themes were identified and reviewed, following the method developed by Braun and Clarke (2006). An abductive coding approach was used (Timmermans & Tavory, 2012), involving an iterative movement between empirical data and existing theory. Initial coding was inductive and closely grounded in participants’ accounts. During this process, several observations were identified that could not be fully explained through inductive coding alone, prompting further analytical engagement with existing theoretical concepts related to POL. In subsequent analytic cycles, concepts derived from the concept analysis of POL by Van Wijk et al. (2022), such as altruism and a high sense of systemness, were used as sensitising concepts to further interpret and refine emerging themes. This abductive approach allowed unexpected patterns in the data to be examined in dialogue with theory, thereby extending understanding of how POL manifests in practice rather than merely applying predefined categories. After the first three interviews, both the first author and the last author performed coding to enhance analytic rigour. Discrepancies were discussed until consensus was reached. Descriptions of the final themes were generated, and illustrative quotes were selected to support the findings.

### Ethical Considerations

Participants were informed about the study and participation was voluntary. Participants were asked to provide informed consent for audio recording and use of their information. Participants were free to withdraw from the study at any time, without giving a reason to do so. This study was conducted according to the principles of the Declaration of Helsinki (World Medical Association, 2013). The study did not fall under the scope of Medical Research Involving Human Subjects Act (WMO) (“Wet medisch–wetenschappelijk onderzoek met mensen,” 1998) and therefore did not require approval from the Medical Ethical Research Committee (METC). Respondents were pseudonymised by randomly assigning names to the participants. Information that could lead back to individuals was excluded, accordingly with the Dutch Act on Implementation of the General Data Protection Regulation (UAVG).

### Methodological Decisions

To enhance validity, reliability and rigour, the primary criteria of Whittemore et al. (2001) were taken into account. The interview guide was used to collect similar types of data from all participants, resulting in reliability, with consistent results (Holloway & Wheeler, 2010). Member check, for which transcripts were e-mailed to the participants, took place to enhance trustworthiness (Birt et al., 2016). When describing themes, illustrative quotes are provided to support theme descriptions and enhance transparency (Holloway & Wheeler, 2010), producing a scholarly report of the analysis (Braun & Clarke, 2006). The study is reported accordingly with the COREQ Criteria (Tong et al., 2007). Quotes have been translated and checked by a bilingual editor to preserve meaning.

## Results

Twelve Dutch nurses, who raised their voices in public media on the proposed law “BIG2”, were interviewed. Eight of the participants were female and four were male. The median age of the participants was 46,5 years with work experience ranging from 7 to 42 years. All but one participant had work experience in a hospital. In addition, three participants (25%) had experience in non-bedside roles. The group represented a variety of educational backgrounds, including DN (50%), VN (25%) and BN (58%). Several participants had completed more than one level of education. In addition, most participants (75%) held a clinical specialty, and a smaller number (17%) had obtained a Master of Science degree (Table 2).^1^

**Table 2.** Baseline Characteristics.

Characteristic	Category	N (%)
Age category	21–30 years	1 (8,3%)
	31–40 years	3 (25%)
	41–50 years	4 (33,3%)
	51–60 years	2 (16,7%)
	61–70 years	2 (16,7%)
Educational background	Diploma Nursing	6 (50%)
	Vocational Nursing	3 (25%)
	Bachelor of Nursing	7 (58,3%)
	Clinical specialty	9 (75%)
	Master of Science	2 (16,7%)
Work experience	Bedside	12 (100%)
	Non-bedside	3 (25%)
Fields of experience	Hospital	11 (91,7%)
	Nursing home care	2 (16,7%)
	Education	1 (8,3%)
	Insurance	1 (8,3%)
	Policy	1 (8,3%)
Years of experience – Hospital	1–10 years	1 (8,3%)
	11–20 years	5 (45,5%)
	21–30 years	2 (18,2%)
	31–40 years	1 (8,3%)
	41–50 years	2 (18,2%)
Years of experience – Nursing home care	1–10 years	2 (100%)
Years of experience – Education	1–10 years	1 (100%)
Years of experience – Insurance	1–10 years	1 (100%)
Years of experience – Policy	11–20 years	1 (100%)
Opinion	In favour (pro)	6 (50%)
	Opposed (con)	6 (50%)

N = 12 Respondents. *Response options were not mutually exclusive.*

To elaborate on which types of behaviour the nurses engaged in during their public voice, the results first outline the kinds of public activities participants undertook and the situations in which this behaviour was shown. Next, three motivational processes of nurses are described that led to nurses engaging in such public activity, followed by the ways in which they tried to increase their public impact.

### Public Behaviour Expressed by Nurses

The participants engaged in a range of public activities to express their views on the proposed law. They wrote opinion articles and letters in national newspapers, appeared on television and radio programmes, and contributed to professional journals. Several nurses shared their perspectives in online discussions or social media groups, often combining traditional and digital media to reach broader audiences. In these activities, they positioned themselves as both representatives of everyday nursing practice and as professionals seeking to clarify misunderstandings about their work.

### Professional Identity as Foundation for POL

The participants, nurses who acted as public opinion leaders during the BIG2 debate, shared several common characteristics. All participants had a clinical background and many years of experience in patient care. They spoke from the perspective of daily practice rather than from formal leadership positions. Their engagement was grounded in professional credibility: they felt entitled to speak because they knew the realities of nursing work.

Some participants entered the debate with prior experience in communication and media, which made them more confident and effective in approaching journalists and shaping their message. One of them explained “*I’ve always been interested in media, I knew how media works and what a press release must look like to make it unique.”* Others developed such skills along the way, learning how to write opinion pieces or express their ideas clearly.

In summary, the nurses showing POL were experienced clinicians acting from moral belief, whose professional expertise and communicative ability helped them take a visible role in the public debate.

### Types of Motivations to Engage in Public Debate

#### Feeling Misunderstood

A main reason for speaking up was the feeling that the nursing profession was misunderstood or misrepresented in the proposed law. Participants described emotions such as frustration, anger and indignation about how nursing was portrayed. These feelings acted as triggers for action. One nurse explained *being “angry at a minister who really had no idea what he was talking about”* Another explained, “*It is ridiculous what happens here, what I still feel is not only the indignation, but there is less and less appreciation.”* Such experiences of being misunderstood motivated nurses to correct misconceptions and to restore the image for the nursing profession.

#### Feeling Responsible for the Nursing Profession

Another motivation was a strong sense of responsibility towards the nursing profession. Some participants felt uniquely positioned to act because of their background, for example, having worked as both vocationally and bachelor-trained nurses. This dual perspective gave them the feeling that they had to contribute and clarify the differences between these roles. As one participant explained, “*If anybody can speak about the difference, it would be me. I worked as vocationally trained nurse for more than eight years before I started my baccalaureate education. So I can use my own experience to indicate the differences.”*

This sense of duty extended beyond personal experience: participants saw their involvement as representing colleagues. Speaking up was a way to act on behalf of the broader nursing community.

#### Acting in Solidarity

Solidarity also played a role in motivating nurses to become publicly active. Seeing other nurses take a stand created a sense of belonging and collective strength. For several participants, this feeling of not being alone reduced hesitation and gave them the confidence to speak out. One nurse described how support from colleagues made participation feel safer: “*I was supported by others, and another nurse joined as well, so I didn’t feel standing alone in this. We were together.”* Solidarity, in this sense, functioned as an emotional trigger: joining others who shared the same concern made public engagement feel both safer and more meaningful.

#### Ways of Increasing Public Impact

Once nurses had become publicly active, they also took steps to strengthen their collective influence. One way of doing so was by turning solidarity into action: actively encouraging colleagues who were hesitant to participate, and reassuring them that they would be supported. As one participant shared, they said: “*We would really appreciate you joining us, we will stand by you.”*

Another strategy involved building and maintaining networks of like-minded nurses. Participants used social media platforms such as Facebook to exchange ideas and experiences, forming online communities that provided both visibility and mutual support. Some nurses also reached out directly to politicians by email or through social media, to raise awareness of their concerns. They noticed that political responsiveness increased when issues gained media attention, suggesting that media visibility and political engagement reinforced each other. As one nurse described, “*we visited all political parties with a small group, that really had effect. And when it gains media attention, you notice that they are all over it as well.”*

These actions demonstrate that POL among nurses was sustained through collective effort, communication, and mutual support. These elements form the foundation for understanding how POL takes shape in practice.

## Discussion

This study explored how POL manifested among nurses who actively engaged in the public debate surrounding the BIG2 proposal, revealing leadership as an expression of moral conviction and professional credibility rather than formal authority. The findings show that emotions, responsibility, and solidarity were central in motivating nurses to speak up, while communication skills and networks helped sustain their engagement over time.

A key feature of nurses’ leadership was their strong sense of moral and professional responsibility. Participants emphasised that their engagement was not driven by personal ambition but by a wish to protect good nursing care and represent their colleagues, as well as patients. This reflects a strong sense of moral responsibility: participants felt they had to defend what they saw as good nursing care. The findings confirm and bring to life the attributes described by Van Wijk et al. (2022), such as clinical expertise, moral commitment, and strategic action. Rather than repeating theory, this study shows how POL actually takes shape in practice: through nurses who speak from lived experience and ethical conviction.

Emotions such as frustration, anger, and indignation were key triggers for action. These were not just expressions of annoyance, but reactions to feeling misunderstood or undervalued. Nurses spoke up to correct that image and to show the real value of their work. This finding aligns with Cummings et al. (2018), who describe how emotional engagement helps leaders connect authentically with others. In this study, emotions transformed dissatisfaction into constructive action, illustrating how affect can energise collective advocacy.

A second mechanism that sustained nurses’ engagement was the combination of collective responsibility and solidarity among nurses. Many described feeling compelled to speak up because of their background or dual training, while others were encouraged by colleagues who promised support. Solidarity turned hesitation into collective action and helped sustain engagement over time. This dynamic shows how POL is not only an individual attribute but also a relational process grounded in shared purpose and mutual reinforcement.

Dynamic networking was central to how nurses amplified their influence. Through social media and personal contacts, they connected with peers, journalists, and politicians. Social media acted as both a bridge and a battleground: it made leadership visible and interactive, as recognized by Van Wijk et al. (2025b), but also exposed nurses to harsh reactions and personal attacks. This dual effect reflects the reality of leadership in the digital era. As Pfister (2011) describes, the constant flow of online discussion can spread ideas quickly but can also lead to polarisation. Álvarez-Muñoz et al. (2025) similarly highlight that professional nursing organisations often still lack clear communication strategies, leaving individual nurses to handle these challenges on their own. The participants in this study could therefore be seen as political advocates: professionals who used media and policy channels to represent their field and raise issues that mattered to nursing. Mason and Ricciardi (2025) argue that communicating with the public and the media is a professional responsibility for nurses, and that increasing visibility in public discourse is key to ensuring nursing expertise is recognised in health policy. This study illustrates how such visibility can develop organically when nurses turn moral conviction into collective action and use communication networks to bring their professional insights into the public debate.

Shared moral values, especially the idea of “doing it for the patient,” were central to how nurses explained their actions. This reflects what Downs and Fiore-Lopez (2022) describe as a core element of nursing advocacy. At the same time, shared moral language can also mask disagreement. As Den Uijl and Van Twist (2018) note, common values can unite people but also mask deeper tensions. In the BIG2 debate, both those in favour of and those against role differentiation, used “doing it for the patient” to support their arguments. This shows how shared values can both connect and divide a professional group, depending on how they are interpreted and mobilised. From a theoretical perspective, these findings suggest that POL in nursing should be further understood as a collective and relational process rather than an individual trait or formal role. Future research could build on this insight by examining how POL develops over time, how nurses who are less visible or hesitant engage with public debates, and how organizational or educational contexts shape opportunities for POL. Comparative studies across policy issues or national contexts may further illuminate how moral conviction, professional credibility, and collective support interact under different conditions. Such work could contribute to refining POL as a dynamic concept that captures both individual motivation and collective action in contemporary nursing practice.

### Limitations and Strengths

This study has some limitations that should be acknowledged. First, due to COVID-19 restrictions, a few interviews were conducted by telephone rather than face to face. Although this limited the possibility of observing non-verbal cues, participants were experienced communicators who appeared comfortable sharing their views. The depth and openness of the conversations did not seem affected. Second, the study focused intentionally on nurses who had already expressed their views publicly. This purposive sampling approach ensured that all participants had direct experience with POL. However, it also means that the findings represent the perspectives of nurses who were already active and comfortable with public engagement, rather than the broader nursing population. Future research could include nurses who are less visible in public discussions to explore how POL is perceived or developed across different professional contexts. Third, participants were not separated into groups based on their opinion on role differentiation. Analysing these subgroups could further highlight whether perspectives on the policy issue influence the way POL is expressed.

Despite these limitations, the study has several notable strengths. To ensure transparency and rigour, the research process followed the COREQ criteria (Tong et al., 2007). These guidelines informed the design, data collection, and reporting of the study. Consistency across interviews was supported by a structured interview guide (Holloway & Wheeler, 2010). Member checking was conducted by sharing transcripts with participants (Birt et al., 2016). Their feedback confirmed that their experiences and perspectives were reflected correctly, which strengthened the credibility and trustworthiness of the results. The analytical approach combined inductive thematic analysis (Braun & Clarke, 2006) with abductive reasoning (Timmermans & Tavory, 2012), allowing data and theory to inform each other iteratively. This approach helped capture both the concrete experiences of nurses and the broader conceptual patterns underlying POL. Together, these methodological choices contributed to a nuanced and empirically grounded understanding of the phenomenon.

### Recommendations

Based on these findings, several recommendations can be made for nursing practice, organisations, and education: (a) Support collective nurse voices. Nurses who speak up often act from a sense of responsibility and a wish to protect good nursing care. Including them in conversations about professional and policy issues helps ensure that decisions reflect the realities of nursing practice, reflecting the idea of “*nothing about us, without us.” (*Buurman, 2020*)*; (b) Create space for mutual support. Many participants described that encouragement from others made it easier to act. Making room for informal conversations or peer support around speaking up could help nurses feel confident and connected when doing so; and (c) Address the role of social media explicitly. Social media played a central role in enabling nurses to express leadership, but it also introduced challenges. Creating structured opportunities within teams, organizations, or educational programs to reflect on social media use may help nurses navigate visibility, influence, and vulnerability when engaging publicly. Discussing both opportunities and risks can help nurses use these platforms more consciously and safely.

Public opinion leadership is not about hierarchy or formal authority, but about nurses who speak from experience and moral conviction. When their voices are supported and connected, they can influence not only how others see the profession, but also how nursing shapes the future of health care.

## Conclusions

This study explored how POL manifested among Dutch nurses who took part in the public debate on the proposed BIG2 health care reform. POL appeared as a form of leadership grounded in moral conviction, clinical expertise, and the willingness to speak up for the profession. Emotions such as anger and frustration were important triggers, typically arising from feeling misunderstood or undervalued; responsibility, solidarity, and encouragement helped sustain engagement.

By using media and professional networks, nurses made their leadership visible and interactive, translating personal experiences into collective advocacy. POL therefore represents a way for nurses to extend their influence beyond the clinical setting and contribute to public and policy discussions about health care.
